# A 16-Year Analysis of Aesthetic Surgery Volume and Its Association with U.S. Economic Performance

**DOI:** 10.1093/asjof/ojae007.084

**Published:** 2024-04-12

**Authors:** Caroline Bay, Peter Wirth, Ellen Shaffrey, Sarah Thornton, Venkat Rao

**Affiliations:** Baylor College of Medicine, Houston, TX; University of Wisconsin, Madison, WI; University of Wisconsin, Madison, WI; University of Wisconsin, Madison, WI; University of Wisconsin, Madison, WI

## Abstract

**Goals/Purpose:**

Historically, demand for plastic surgery has been associated with the performance of the United States (US) economy. Over the past two decades, the US has weathered a recession, several presidential election cycles, and has seen a rise in the use and influence of social media. Each of these events has led to varying effects on the economy, which undoubtedly have had an impact on plastic surgery demand and expenditures. Thus, this study aims to evaluate the relationship between indicators of economic performance and the popularity and profitability of aesthetic surgery from 2006 to 2022.

**Methods/Technique:**

Data from the Aesthetic Society’s (AS) Aesthetic Plastic Surgery National Databank and the American Society of Plastic Surgeons’ (ASPS) Plastic Surgery Statistics Report was collected from 2006 to 2022. Surgical procedures analyzed included the most commonly performed cosmetic surgeries: breast augmentation, breast reduction, mastopexy, abdominoplasty, liposuction, blepharoplasty, facelift, and rhinoplasty. The non-surgical botox injections and dermal fillers were also included. A total of 24 variables were examined, including the 10 procedures and two composite variables—total surgical procedures and total injectables; case change percent difference and patient expenditures percent difference were recorded for each of these. Within the corresponding period, economic indices were collected, including personal disposable income per capita (PDI), consumer price index (CPI), medical care services CPI, average gross domestic product per capita (GDP), and annual average closing prices of the NASDAQ, S&P 500, and the Dow Jones (DOW). Pearson correlation tests were used to analyze the strength of association between each financial indicator and case volumes and expenditures for each procedure included in the ASPS and AS reports.

**Results/Complications:**

From 2006 to 2020 ASPS data demonstrated GDP year-over-year (YOY) change that was positively correlated with case volume and expenditures across 13 out of the 24 different procedure metrics (54.2%). From 2006 to 2016, AS data was positively correlated with the performance of NASDAQ, S&P500, and DOW in 12 of the 24 procedure metrics (50%). GDP YOY change closely followed suit with positive correlations to 11 variables (45.8%).

YOY change of PDI, CPI, and medical care services CPI were less frequently associated amongst both data sets. For ASPS data, YOY medical services CPI change was negatively associated with five variables, indicating that as medical services CPI increased, volume of procedures and expenditures decreased. PDI YOY change was not significantly associated with any variables. In the AS dataset between 2006 and 2016, CPI YOY change was not found to be significantly associated with any variables. Across 2019–2022, CPI YOY and disposable YOY change were found to be significantly associated with three variables each.

**Conclusion:**

Our study suggests that aesthetic plastic surgery procedures and expenditures are most positively correlated with GDP. Stock market indices are additional financial indicators which have significant positive correlations with plastic surgery case volume and expenditures. Inflation indicators were less frequently correlated. Over this timeframe, several important financial events have occurred including the Great Recession, a stock market selloff in 2015 and 2016, and the COVID-19 pandemic. Each of these events, along with several election cycles and the increasing influence of social media and popular culture, have had variable impacts on consumers and the demand for aesthetic plastic surgery. Plastic surgeons should be familiar with US and global financial markets as they fluctuate with current events. While plastic surgery prices and demand may be difficult to anticipate, this study elucidates several factors that may be used by plastic surgeons as a bellwether for their practice.

